# Effects of Different Inter-Row Soil Management and Intra-Row Living Mulch on Spontaneous Flora, Beneficial Insects, and Growth of Young Olive Trees in Southern Italy

**DOI:** 10.3390/plants11040545

**Published:** 2022-02-18

**Authors:** Giuseppina Las Casas, Corrado Ciaccia, Valeria Iovino, Filippo Ferlito, Biagio Torrisi, Enrico Maria Lodolini, Alessio Giuffrida, Roberto Catania, Elisabetta Nicolosi, Salvatore Bella

**Affiliations:** 1 Consiglio per la Ricerca in Agricoltura e l’Analisi dell’Economia Agraria (CREA)—Centro di Ricerca Olivicoltura, Frutticoltura e Agrumicoltura, Corso Savoia 190, 95024 Acireale, Italy; giuseppina.lascasas@crea.gov.it (G.L.C.); iovino.valeri@gmail.com (V.I.); biagiofrancesco.torrisi@crea.gov.it (B.T.); alessio020594@live.it (A.G.); robertocatania1995@libero.it (R.C.); salvatore.bella@crea.gov.it (S.B.); 2 Consiglio per la Ricerca in Agricoltura e l’Analisi dell’Economia Agraria (CREA)—Centro di Ricerca Agricoltura e Ambiente, Via della Navicella 4, 00184 Roma, Italy; corrado.ciaccia@crea.gov.it; 3 Consiglio per la Ricerca in Agricoltura e l’Analisi dell’Economia Agraria (CREA)—Centro di Ricerca Olivicoltura, Frutticoltura e Agrumicoltura, Via Fioranello 52, 00134 Roma, Italy; 4 Dipartimento di Agricoltura, Alimentazione e Ambiente (Di3A), Università di Catania, Via Valdisavoia 5, 95123 Catania, Italy; elisabetta.nicolosi@unict.it

**Keywords:** *Olea europaea* L., Mediterranean basin, agroecological practices, minimum tillage, zero tillage, pollinating and predatory insects, agroforestry, intercropping, consociation

## Abstract

Conservation agriculture (i.e., minimized soil disturbance and permanent soil covering) and living mulches represent two agroecological practices that can improve soil fertility, spontaneous flora, and beneficial insect communities. This research studied the effect of these practices in a young olive orchard in the Mediterranean area. Two Sicilian olive cultivars (‘Nocellara del Belice’ and ‘Nocellara etnea’) were used for the field experiment; inter-row minimum and zero tillage and four species of aromatic plants as living mulch along the row were tested. Spontaneous flora and beneficial insect communities, as well as tree growth, were monitored. The inter-row management did not influence the spontaneous flora dynamics. The species adopted for living mulch showed a very different degree of development and soil cover; 69 insect species (pollinators and predators) belonging to five orders (Hymenoptera, Lepidoptera, Diptera, Neuroptera, and Coleoptera) and 17 families were recorded. The growth of the olive trees was not affected by the conservative strategies.: In the inter-row, the growth of the spontaneous flora was limited by the high temperatures during the summer. Among the living mulch species, sage and lemongrass guaranteed an almost full soil cover, reducing the need for weed management along the row, as well as increasing the beneficial insects without influencing the young tree growth.

## 1. Introduction

One of the main goals established by the European Commission during the period 2019–2024 is to lay the foundation for making the European Union the first climate-neutral continent by 2050. To achieve this objective, the Commission presented the European Green Deal policy, the most ambitious package of measures that should enable European citizens and businesses to benefit from sustainable green transition. Concerning the agricultural sector, this objective will be reached by a drastic reduction in farm input (fertilizers, chemical pesticide, hormones), reducing the nutrient losses and preserving and restoring ecosystems and biodiversity [1].

Currently, this policy is mandatory considering the ongoing climate change and its impact on agriculture (increase in average temperature and risk of extreme natural events such as floods and droughts) and the land degradation process (erosion, salinity, soil borne diseases) occurring in large areas of the world and the subsequent loss of biodiversity. Moreover, it is important to consider that the world population will increase in the same period (2050) and will reach about 9.1 billion people [2], consequently increasing the food demand [3]. Therefore, in this scenario, agricultural sectors also need to increase the crop efficiency, since the land availability and productivity will play a central role for the maintenance of several rural contexts [4].

The Mediterranean basin is a representative area in which the abovementioned criticisms are well recognized. Among fruit tree crops, the olive (*Olea europaea* L.), one of the most cultivated species that covers about 9.5 million hectares in Europe [5], is an important crop for its social, economic, and ecological role [6].

Regarding the social aspect, it is able to contrast the depopulation of the countryside, as well as maintain the historical aspect of its cultivation [7], providing healthy and safe food for the population. In addition, olive cultivation connects different generations because most of the farmers cultivating traditional olive orchards are aged or retired people, who are still active in agriculture and share their knowledge with younger people in order to maximize the production only using the potential of the agroecosystem [8].

The economic role is well documented; in fact, the olive production has increased in recent years due to the introduction of new planting models [9], mechanization of some cultural practices, harvest above all [10], precision management technologies [11,12], and the use of high-quality standard propagation material [13]. Moreover, at least 95% of the olive cultivation is located in the Mediterranean basin [14], and it represents about 70% of world’s olive production [15], from about 1.9 million olive-growing farms.

In terms of the agroecological value, olive plays a fundamental role in maintaining some fragile areas, preventing soil erosion, as well as loss of water and nutrients, and increasing biodiversity. Moreover, thanks to its historical aspect and adaptation, compared to the other woody crops, olive cultivation does not require high external inputs, thus contributing to reducing environmental pollution [16]. On the other hand, tillage (full or partial) is often realized, while minimum and zero tillage is less adopted. Low-intensity tillage leads to an increase in the number of beneficial insects such as pollinators [17] that sustain wild plant communities providing key ecosystem services (e.g., contributing to control pest and crop disease) [18]. As demonstrated by different studies, various anthropogenic factors, such as the expansion of agriculture and livestock, habitat fragmentation, and irrational use of pesticides and pollution, are causing a global decline in insects [19]. In Europe, 9% of butterflies and 9.2% of wild bees are threatened by conventional agriculture [20]. Conservative agriculture (e.g., minimized soil disturbance, permanent soil covering) has been shown to have an impact on biodiversity and ecosystem service provision [21], reducing the negative effects of conventional tillage and enhancing the number of beneficial insects, as well as improving their role in the agroecosystem. Similarly, diversification strategies in space and time by the inclusion of agroecological infrastructure in agricultural landscape such as hedgerows and cover crops (including living mulch) are considered redesign strategies able to magnify the role of agro-biodiversity in ecosystem service provision [22,23]. Moreover, the management strategies can impact the spontaneous flora community (i.e., the weeds), reducing the selection of competitive flora toward a more service provision-oriented community, by supporting pollinators or beneficial attraction [24]. Then, the introduction of herbaceous species (e.g., intercropping, living mulch) that are not directly aimed at production but provide ecological services, called agroecological service crops (ASC) [25], can positively influence the overall ecosystem functioning by providing pest control and ecological services such as weed control in the row [26], protection of the soil from degradation, an increase in organic carbon content, which improves the soil structure and fertility [27], and a decrease in the concentration of CO_2_ in the atmosphere if properly managed [28]. Among the conservative soil management strategies, consociations (annual or perennial intercropping), soil management practices (minimum tillage, zero tillage), and organic fertilization were considered for a comprehensive meta-analysis (187 experiments realized in the Mediterranean basin with several woody crops for a total of 46 papers) [29] that highlighted a general positive effect of the abovementioned strategies in carbon sequestration compared to mono-cropping, conventional tillage, and inorganic fertilization. For olive, since the last century, consociations with herbaceous or woody species have been described [30]. These were due to the extensive olive orchards, as well as the consociations with livestock where possible [31]. For other species such as grapevines, minimum or zero tillage is commonly applied in order to regulate the vegetative and reproductive balance of vines and, in some cases, in order to reduce erosion and land degradation [32,33,34].

In this context, olive could represent an important source of ecological interest among the numerous Mediterranean species due to its specific characteristics, such as high drought resistance, low chill unit requirement, adaptation to hot and dry climatic conditions, and low pest and disease incidence, all of which are significant characteristics to consider in the establishment of new orchards with an agro-ecological approach [35]. However, it is important to consider that the cultivation of olive trees is very diversified among the Mediterranean countries, and that the social, economic, and agroecological value of the olive orchards is strongly variable according to the different cultivation systems (traditional, intensive, and super-intensive orchards), farming techniques, and genetic resources [36]. In traditional orchards, the social and agroecological characteristics are highly relevant, whereas, in the intensive model, only the olive agroecological importance is essential. In these categories, olive models are in accordance with the main objectives of the agroecological approach, which aims to reinforce the natural strength of the agroecosystem without using external inputs and augment the resilience of the crops, encompassing the social, ecological, and economic dimensions of sustainability [37]. In the super-intensive growing system, the economic factor is of greater importance than the social and agroecological factors.

In our research, we tested the impact of some agroecological practices (i.e., conservative soil management and ASC living mulch introduction) on the wild agro-biodiversity (weed and arthropod communities) and vegetative growth of a newly planted olive orchard. We assumed that different floor management (minimum tillage vs. zero tillage) and intra-row management (different living mulch species vs. no living mulch) would differently influence the dynamics of the monitored agro-biodiversity and the young plant response. In particular, we hypothesized that (i) the zero-tillage floor management would guarantee permanent soil cover without selecting higher competitive flora, (ii) the living mulches would positively influence the presence of beneficial insects, and (iii) different living mulches would have a different impact on both arthropods and weed communities, depending on the introduced species.

## 2. Results

### 2.1. Entomological Report

The complete list of the 69 recorded species of beneficial insects, as well as their relation to the spontaneous flora or the consociated ones, in the studied olive orchard is reported in Table 1 and Table 2. Specimens of pollinators (61 species) and predators (eight species) were collected in the 2 years of field surveys on the wild and cultivated plants. Regarding pollinators, the 33 species of Apoidea reported belong to five different families, Colletidae (one species), Andrenidae (seven species), Halictidae (four species), Megachilidae (five species), and Apidae (16 species), and 15 genera (Table 1). Most of these species nest by digging into the ground (24 species, 72.72%), while 21.21% (seven species) of the taxa nest in pre-existing cavities in the ground, in the walls, or in dry and hollow vegetables. Two species among the 33 observed (6.06%) belong to the *Nomada* Scopoli genus of brood parasitic bees characterized by the presence of females that lay eggs in the nest of other wild bees. Regarding the behavior, 24 species are solitary (72.72%), five species (15.15%) exhibit a pre-social behavior, two species (6.06%) have a social behavior, and two species (6.06%) are brood parasite species.

The 23 species of Lepidoptera reported belong to nine different families, Sphingidae (three species), Sesiidae (one species), Geometridae (two species), Noctuidae (two species) Hesperiidae (one species), Lycaenidae (two species), Nymphalidae (five species), Papilionidae (two species), and Pieridae (five species), and 17 genera (Table 1).

Five species (and five genera) of Diptera were found belonging to the Syrphidae family. The adults of these species are pollinators of spontaneous plants; however, the larvae have different trophic regimes. For example, larvae of *Episyrphus balteatus* (DeGeer), and *Eupeodes luniger* (Meigen) are predators of aphids, while those of *Eristalinus taeniops* (Wiedemann), *Eristalis tenax* (L.), and *Syritta pipiens* (L.) are scavengers [38].

Furthermore, regarding predator insects, two species of Neuroptera Chrysopidae and six species of Coleoptera Coccinellidae were found; among these, one species feeds mainly on coccids, while the others feed mainly on aphids.

### 2.2. Spontaneous Flora Distribution and Diversity

The complete list of the spontaneous flora species found in the field, as well as the time (spring or autumn) and the area in which they were recorded (inter-row or intra-row), is reported in Table 3. A total of 40 species of plants are listed. Among these, five species, *Amaranthus retroflexus* L. (AMARE), *Cynodon dactylon* (L.) Pers. (CYNDA), *Cyperus rotundus* L. (CYPRO), *Polygonum aviculare* L. (POLAV), and *Portulaca oleracea* L. (POROL), were found in each period and position. In spring, 28 species of plants were detected: 14 of them both in the intra-row and inter-row, and the remaining 14 exclusively in the intra-row. On the contrary, no exclusive species in the inter-row were observed. In autumn, 26 species were observed, and only eight grew both in inter-row and intra-row. In this period, six species were exclusive in the inter-row while 11 species were found only along the row.

Regarding the weed monitoring achieved in spring in the inter-row, in MT treatments, most of the space (70%) was classified as bare soil, while the predominant spontaneous plants were *Portulaca oleracea* (POROL) (11%) and *Convolvulus arvensis* L. (CONAR) (7%), even though the quantity was lower compared to the ZT treatment. In these areas, the bare soil was in less quantity (18%), and the predominant spontaneous plant was *Papaver rhoeas* L. (PAPRH) (almost 40% of the total space was occupied from this plant), followed by *Beta vulgaris* L. (BEAVX) (almost 25%).

In terms of the distribution of the weed community during spring in the intra-rows, in the MT treatment, the prevalent species found were *Portulaca oleracea* (POROL) (13%) and *Cynodon dactylon* (CYNDA) (10%), while the remaining weeds showed a distribution more or less constant along the intra-rows. Regarding the frequency and distribution of the spontaneous flora community in the intra-rows in ZT treatments, *Papaver rhoeas* (PAPRH) was present in a larger proportion (27%) compared to the others, followed by *Beta vulgaris* (BEAVX) (10%). The presence of other weed species was similar to that observed in the tillage blocks even if, in the control, *Papaver rhoeas* (PAPRH) covered about 60% of the soil.

In the autumn survey, it was observed how the vegetation developed almost exclusively along the rows due to the presence of irrigation, whereas, in the inter-row, a high percentage of bare soil (MT 96%; ZT 77%) was registered. Along the row, there was a significant increase in the space occupied by ASC species, particularly sage and lemongrass, and, for both MT and ZT, the most represented spontaneous species was *Setaria verticillata* (L.) P. Beauv. (SETVE).

**Figure 1:**
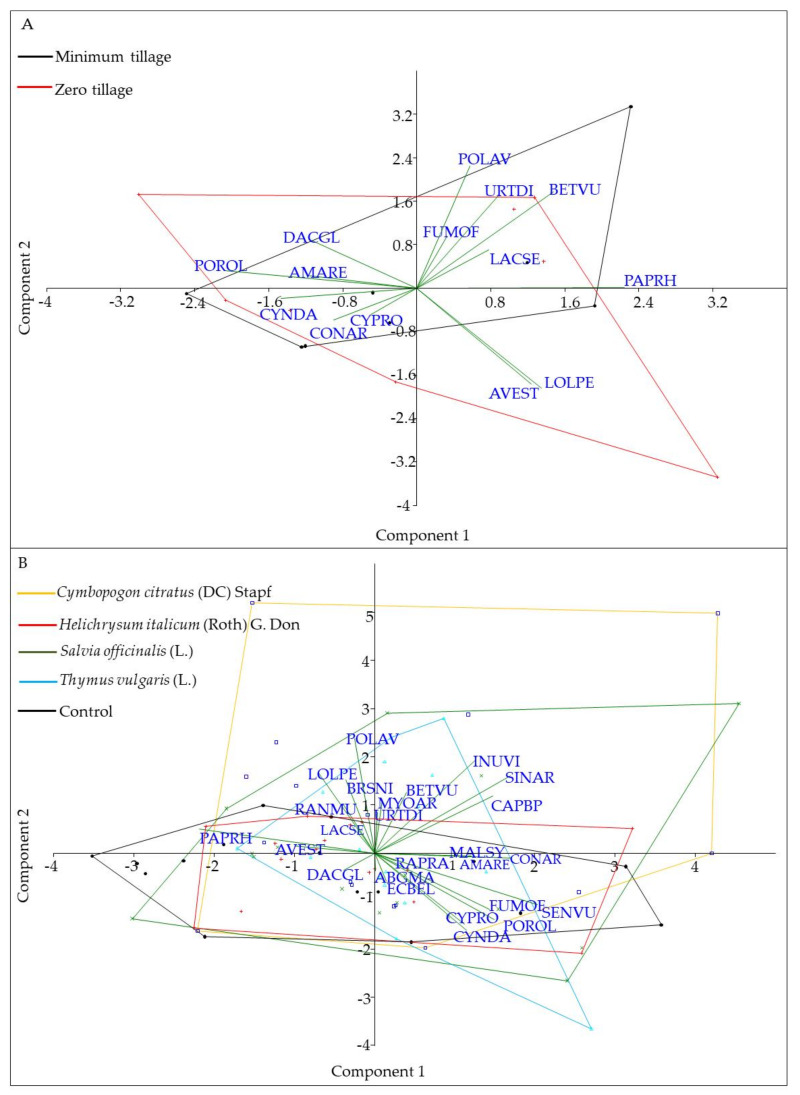
(**A**,**B**) Principal component analysis (PCA) ordination diagram (biplot) depicting the localization of the studied samples from the experimental trial in relation to the inter-row (**A**) and intra-row (**B**) management.

**Figure 2:**
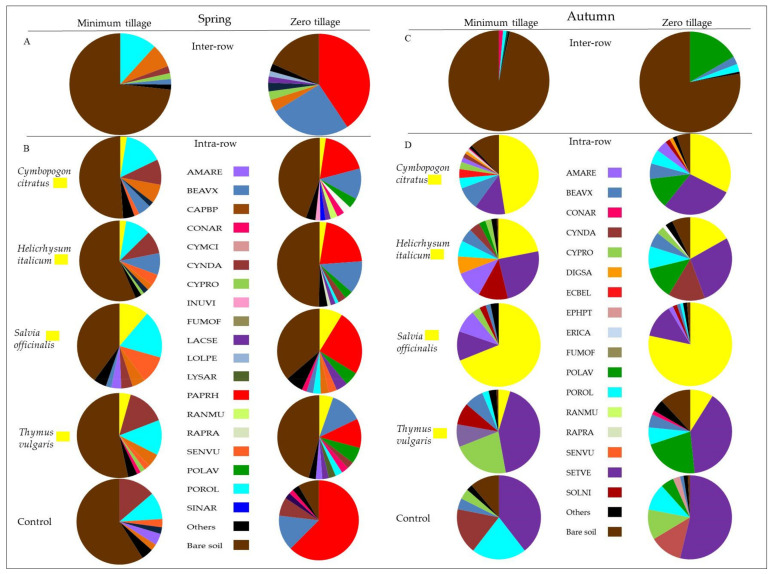
Spontaneous flora species covering percentage in spring (**A**) and autumn (**C**) over the inter-row and along the intra-row (**B**,**D**) of the experimental field ‘long-term trial on organic olive (BiOlea)’.

A principal component analysis (PCA) was carried out to evaluate the effect of the ASC on the development, quantity, and distribution of the weed community. With respect to soil management data analysis, Component 1 explained 20.97% of the total variability, while Component 2 explained 15.71% (Table 4). According to the PCA results relative to the spring and autumn analysis, as shown in Figure 1A,B for the spring stage, there were no significant differences in terms of distribution between the plots analyzed. Regarding the distribution of the spontaneous flora community in the inter-row with different soil management (ZT and TI) (Figure 1A), weed species appeared divided into four main groups (Figure 1A) characterizing the community: perennial species (namely CONAR, CYNDA, and CYPRO), AMARE, POROL, and DACGL (group 1) were negatively correlated to POLAV, URTDI, BETVU, FUMOF, and LACSE (group 2), and PAPRH (group 3), whereas two completely independent grass species appeared, AVEST and LOLPE (group 4). Despite this, PCA did not show clear differences in terms of abundance and distribution. On the other hand, the zero-tillage community was characterized by the presence of AVEST and LOLPE, whereas BETVU (BEAVX) and URTDI showed a higher relationship with minimum tillage (Figure 2A,C). At this stage (spring), the intra-rows with sage, lemongrass curry plant, and thyme living mulch and control all presented a weed community where all the specimens had an average distribution, with some peak presence of AMARE in sage mulch rows and of SETVE in the control row (Figure 2B,D). These records are an overview of 1 year of the field trial and still need to be re-evaluated in the long term management of the orchard. Similar results were obtained for the second assessment in autumn (not shown).

### 2.3. Plant Growth Analysis

**Figure 3:**
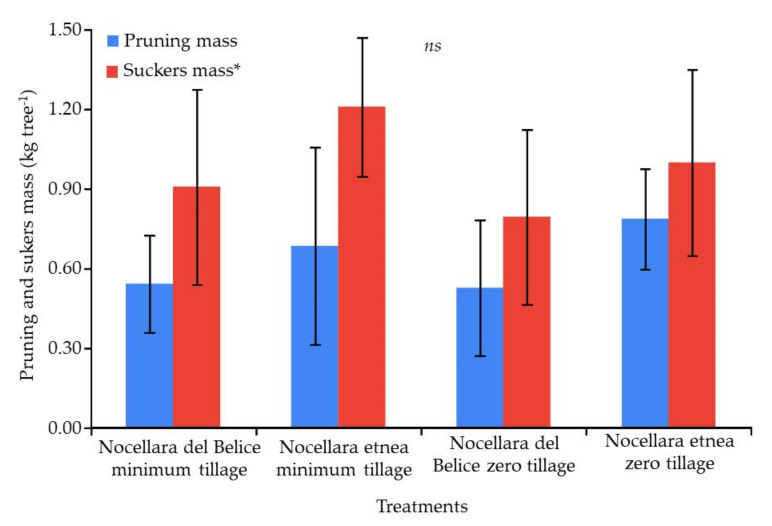
Influence of soil management strategy on winter pruning and sucker mass produced (ns = not significant within each parameter; bars indicate standard deviation) according to Tukey’s HSD test, for each treatment and parameter. * Comprehensive record of the shoots weight grown from the ground level to the branch insertion.

**Figure 4:**
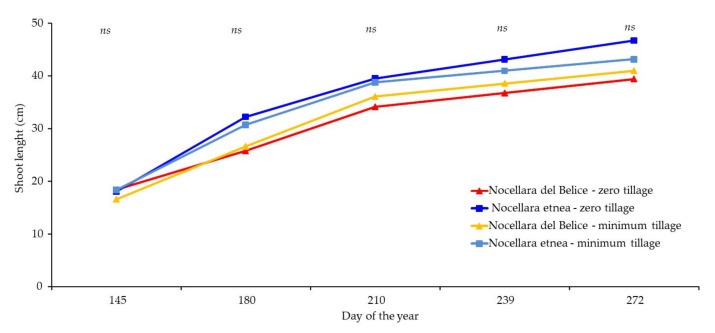
Influence of soil management strategy on mixed shoot growth (ns = not significant) according to Tukey’s HSD test, for each treatment and parameter.

In terms of the produced biomass removed with winter pruning (in February), the most abundant quantity was recorded for the NE cultivar in both soil treatments. In September, the quantity of emitted material (suckers and shoots removed from the trunk) was the highest in NE-MT (Figure 3). Concerning the shoot growth monitoring, despite the absence of significant differences among treatments, a better performance for NE in both soil treatments was observed. In general, the growth rate was about 10–12 cm between day of the year (DOY) 145 and 180, about 8–10 cm between DOY 180 and 210, 2–3 cm between DOY 210 and 239, and 2–3 cm between DOY 239 and 272 (Figure 4). The plant growth response to the applied soil management is reported in Table 5. The trunk cross-sectional area reached the highest growth for both cultivars in the zero-tillage soil management. The canopy height increase (approximately 30%) was similar among treatments, although NE-ZT showed the highest growth. The trunk cross-sectional area (TCSA) showed more variable results, with NE-ZT and NB-ZT showing the highest growth (+105% and 96%, respectively), while NB-TI showed an expansion of about 48% and NE-TI of just 17%. According to the data presented in Figure 4, all variables had the same rate of growth, with an increase of about 10–12 cm between DOY 145 and 180, 8–10 cm between DOY 180 and 210, 2–3 cm between DOY 210 and 239, and 2–3 cm between DOY 239 and 272. This trend is in accordance with the normal development of the olive trees during their young phase, as well as with the climatic data and water intake registered during the trial.

## 3. Discussion

This study focused on three key indicators in agro-ecosystems: (1) the insect community, (2) the spontaneous flora diversity, and (3) the young olive response in terms of vegetative growth. Therefore, in our study, the entire soil–plant–atmosphere *continuum* (SPAC) was analyzed.

The entomological study was performed in terms of both pollinators and natural enemies. The research was conducted in an olive orchard located on a farm in a district with high relevance for citrus and other fruit crops. The collected Apoidea were observed on 23 species of wild plants, comprising a total of 23 plant genera within 16 plant families (Table 1 and Table 2). The Asteraceae family was that frequented by the greatest number of pollinators (15 species), followed by Brassicaceae (12 spp.) and Ranunculaceae (five spp.) (Table 3). On the consociated plants, 39 species of pollinators were observed, 25 on *Thymus vulgaris*, 12 on *Salvia officinalis* (Lamiaceae), and nine on *Helichrysum italicum* (Asteraceae).

Currently, 686 species of bees are known in Sicily [39]. The comparison of bee fauna in the Palazzelli agro-ecosystem evidenced a total of 33 species (4.8% of the species known for the Sicilian fauna) belonging to Colletidae (one species) Andrenidae (seven spp.), Halictidae (four spp.), Megachilidae (five spp.), and Apidae (16 spp.) families.

The order Lepidoptera, the second most important group, was present with 23 species, comprising 16 butterflies and eight moths.

In terms of wild bees, it is significant to note that 72.72% (24 species) of the overall species nest in the ground, and their existence depends on the typology of soil management. In recent years, various regional surveys have focused on the biodiversity of these populations and the agroecological role of these two groups of insects [40,41,42] or as specific pollinators of crops [43,44,45,46].

In order to maintain Apoidea biodiversity, management practices should take into account that most species of wild bees nest in the ground [47], and different agronomic practices, including tillage of the land, usually render crops an unsuitable habitat for wild bees, especially in intensive management [48]. In particular, deep tillage and total removal of spontaneous vegetation represent a serious problem for the foraging and nesting of these pollinators [49]. Therefore, in agricultural environments, wild bees need semi-natural habitats for nesting, obtaining the floral resources, and overwintering. The elements of the landscape, in the field and around the field, also have the function of habitat for fauna in general and, in this context, of ecological corridors in intensely cultivated and biodiversity conservation areas [50,51]. It is also necessary to consider how useful effects are particularly important in Mediterranean agro-ecosystems subject to desertification [52,53,54,55,56].

The consociated plants in the intra-row were visited by 62.3% (43 species) of collected insects, 62.2% of all pollinators and 62.5% of all predators. Overall, 15.9% (11 species) of all reported insects were found only on consociated plants, 16.3% of pollinators and 12% of predators.

In our trial, conservative models were also proposed to increase soil fertility and biodiversity (insects and spontaneous flora in the inter-row), reducing the costs for soil management and improving the spontaneous flora control along the row. Our findings evidence small differences between the two soil management strategies. In particular, minimum tillage showed a higher reduction in weed presence at both sampling times (spring and autumn) as confirmed by the higher bare soil cover than in the zero-tillage system (Figure 3). This result evidence how single tillage is an efficient weed management strategy. On the other hand, ZT showed a higher weed cover than MT and a higher richness (data not shown). Nevertheless, ZT in spring showed the selection of perennial species (namely, CONAR, BEAVX, CYPRO, and LOLPE; Figure 2A,C) and a higher characterization of some grass-like species (AVEST and LOLPE; Figure 2B,D). This result is in line with previous findings on zero tillage as a filter to shift the community toward grassy annual and perennial species [57,58], representing a risk in terms of competition with young orchards.

The living mulches realized along the row showed different effects according to the adopted species. In spring, only sage covered the main portion of the soil, due to its habitus. In autumn, 6 months after planting, the sage showed a complete hedgerow, and the consociated flora was observed just at the ground level under the plants. Similarly, lemongrass, despite forming an almost dense hedgerow, completely prevented weed growth under the plants thanks to its strong tillering ability, while allowing growth between plants. Therefore, these species contributed to creating a wide soil cover before the winter season and improved the soil performance [59]. Thyme and curry plant recorded the lowest growth and showed reduced power for competition with the spontaneous flora. However, in these cases, the spontaneous flora had a role in the preservation of the essences during summer since they covered the little plants and permitted them to survive during this season. Perhaps, for these essences, two growing seasons are required to reach a complete hedgerow. Therefore, in the inter-row, lemongrass and sage reduced the need for further soil management. The adopted living mulches reduced the propagation of weeds without reducing the vigor and growth of olive trees. It is possible to assume that the distance from the trunk of the young olive trees to the plants of living mulch was about 40 cm, and it did not significantly affect the olive growth. It is important to highlight that the irrigation lines played a strong role for both the olives trees and the consociated species. Since the olive trees were young, full irrigation was useful to reach high growth rates as shown by the increase registered in morphological parameters (Figure 3 and Figure 4, and Table 5). Among these, the canopy volume exhibited strong growth. According to our findings, it is possible to hypothesize two drip lines for differentiated irrigation between olive trees and living mulch species. From a practical point of view, in areas with hot and dry summers, planting in the field is possible in autumn or in spring. One plant every 50 cm is enough to boost the growth of the living mulch along the row, but it is important to consider that, after 6 months, the removal of the lines from the row is very difficult; therefore, positioning above the ground level is preferred.

In general, the obtained hedgerows could represent an integrative crop for a secondary income for the farmer, such as food, feed, or industrial products, increasing the resilience of the system to pest incidence and market volatility [60].

## 4. Materials and Methods

### 4.1. Site Description, Experimental Design, and Treatments

The study was carried out between June 2019 and October 2021, in the ‘long-term trial on organic olive (BiOlea)’, of the experimental farm of the Council for Agricultural Research and Economics (CREA), Research Center for Olive, Tree Fruit, and Citrus located at Palazzelli (Lentini district, Syracuse), Sicily, Italy, (latitude 37.17″ N, longitude 14.50″ E, elevation 45 m a.s.l.). The experiment focused on a young olive orchard, planted with two Sicilian main double aptitude olive cultivars ‘Nocellara del Belice’ (NB) and ‘Nocellara etnea’ (NE), grafted onto seedling rootstocks. Trees were planted in May 2019, in north–south-oriented rows, at a spacing of 6 m between rows and 5 m within the row. The adopted training system, since the first winter pruning season (February 2020), was the polyconic vase, aiming to maintain three main branches. Trees were drip-irrigated early in the morning three times per week, from June to September. Irrigation volume scheduling was based on the FAO-56 Penman–Monteith (P–M) approach [61,62], adjusted by the variable crop coefficient (kc) from 0.15 in the first growing season to 0.34 in the second one [63]. Each of the four drippers per tree emitted 2 L·h^−1^, for a total of 8 L·h^−1^, with an operational pressure of 1 bar. Plants were fully irrigated, corresponding to 95–98% of crop evapotranspiration, ET_c_. The electrical conductivity of the water (at 25 °C) was 2.02 dS·m^−1^ and the pH was 7.30. Only organic fertilization was applied at the plantation.

**Figure 5:**
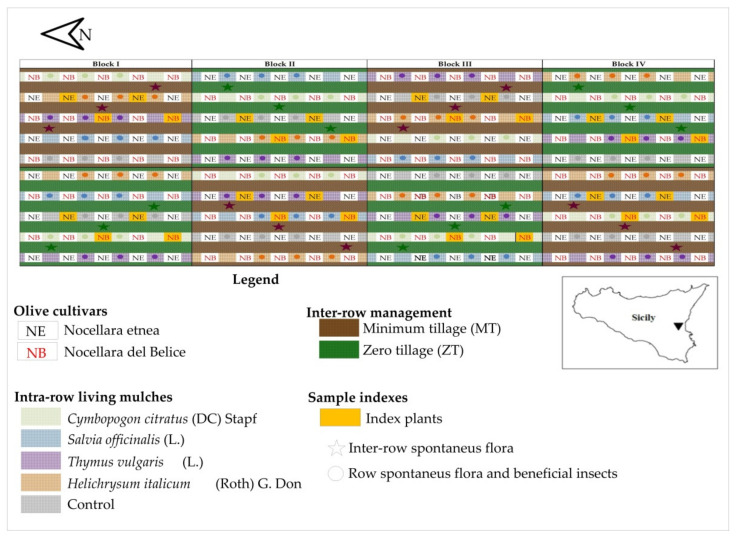
‘Long-term trial on organic olive (BiOlea)’ experimental field design within the experimental farm of the CREA, Research Center for Olive, Tree Fruit, and Citrus located at Palazzelli, Sicily, Italy (latitude 37.17″ N, longitude 14.50″ E, elevation 45 m a.s.l.), with indications of the index plants and the samples points.

The trial was designed as a split-plot system with four blocks of 10 rows with five plants each (Figure 5). The main plot was assigned to soil management practice comparing two systems: (1) minimum tillage (MT) consisting of one tillage (15 cm depth) performed at the end of the winter (first week of March) and (2) zero tillage (ZT) consisting of soil managed only through mechanical shredding, performed twice per year: at the end of the winter, in the same period of MT (first week of March), and at the beginning of summer (four week of June). The sub-plot was assigned to the variety alternating a row with NB and a row with NE, so that compared treatments were (1) Nocellara del Belice—minimum tillage (NB-MT), (2) Nocellara del Belice—zero tillage (NB-ZT), (3) Nocellara etnea—minimum tillage (NE-MT), and (4) Nocellara etnea—zero tillage (NE-ZT).

For the specific activity of this study, on 15 March 2021, a living mulch system was set down along the row using four officinal species as agro-ecological service crops (ASCs) planted at a distance of 0.5 m: (1) sage (*Salvia officinalis* L.), (2) thyme (*Thymus vulgaris* L.), (3) curry plant (*Helichrysum italicum* (Roth) G. Don), and (4) lemongrass (*Cymbopogon citratus* (DC) Stapf). No living mulch between trees along the row was used as control (C), but the spontaneous flora was maintained. Inter-row soil management was used as a factor for field spontaneous flora assessment and for plant growth monitoring in both cultivars. The soil management and the living mulch interactions along the row were used both for the spontaneous flora and for the entomological assessments.

### 4.2. Soil Analysis and Climatic Data

At planting, soil characteristics were analyzed at 20–40 cm depth by three samplings per plot. Soil physical and chemical characteristics are reported in Table 6. Regarding physical characteristics, the quantity and distribution of sand, clay, and silt was obtained by particle-size analysis using the “micro-pipette” method [64]. In terms of chemical properties, total nitrogen (N), organic matter (OM), soil extractable phosphorus (mg/kg), soil exchangeable potassium (meq/100 g), cation exchange capacity, pH, and electrical conductivity (EC) determinations were determined as described in [65,66,67,68,69,70,71]. Total nitrogen was measured by Kjeldahl digestion using a Buchi Labortechnik GmbH N analyzer, and organic matter (OM) was measured by quantifying total organic carbon (TOC, mg·kg^−1^). TOC was analyzed by means of elemental analyzer LECO (RC-612; St. Joseph, MI, USA) using a dry combustion method. Soil exchangeable potassium (meq/100 g) was determined in a solution of barium chloride and triethanolamine at pH 8.2 (2 g of soil: 25 mL). Cationic exchange capacity was analyzed by the BaCl_2_ compulsive exchange method. The pH and EC determinations were carried out on a HI 9813 portable EC meter (Hanna Instruments, Woonsocket, RI, USA) and an AB 15 pH meter (Thermo Fisher Scientific, Waltham, MA, USA), respectively. Inductively coupled plasma optical emission spectrometry, ICP-OES, was conducted using an Optima 2000 DV, PerkinElmer Inc. Shelton, CT, USA). According to the United States Department of Agriculture (USDA) scheme, the olive-grove soil is classified as loamy sand [72]. The soil pH is subalkaline, and electrical conductivity is considered low [73]. 

**Figure 6:**
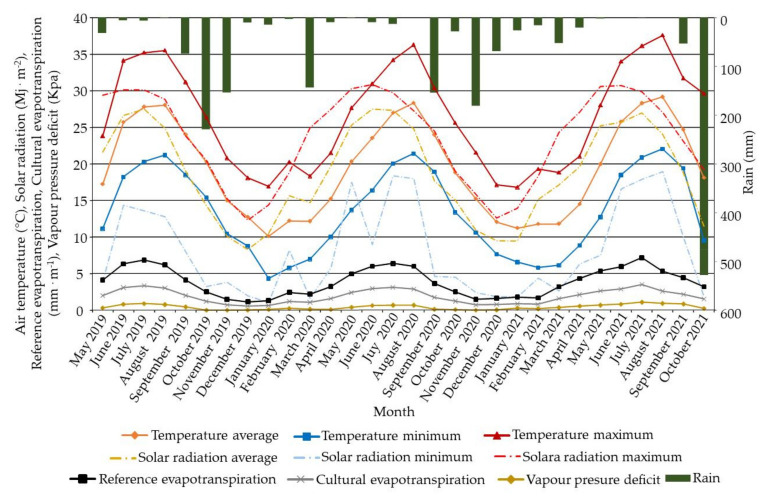
Monthly minimum, average, and maximum air temperature and solar radiation, rainfall, reference and cultural evapotranspiration, and vapor pressure deficit registered in the experimental field ‘long-term trial on organic olive (BiOlea)’.

Climatic data, namely, monthly minimum, mean, and maximum air temperature, global solar radiation, rainfall, reference evapotranspiration (ET_0_), cultural evapotranspiration (ET_c_), and vapor pressure deficit, registered at the experimental field, were collected from an agro-meteorological station located in the experimental farm (Figure 6). The climate of the region is typical Mediterranean, with hot and dry summers. According to the available meteorological data (30 years, not shown), annual mean reference rainfall is about 550 mm, and the maximum temperature in summer during daytime often reaches 38–40 °C [74]. During the trial, the site’s climate was characterized by mild and wet winters, while the summers were semiarid (first and second) and dry (third) in which no rainfall was recorded from May to August. The annual average temperature was 18.29 °C. The lowest minimum temperatures were recorded in January and February. Mean temperature values were always above 22 °C from April to November.

### 4.3. Entomological Samplings and Analysis

Entomological studies, regarding pollinators (Hymenoptera Apoidea, Lepidoptera, and Diptera Syrphidae) and predator insects (Neuroptera and Coleoptera Coccinellidae), were carried out twice per month, from March 2020 to October 2021. In particular, from 1 March 2020 to 28 February 2021, insects were collected from 2500 m^2^ for each of the two soil management areas (125 m^2^ each inter-row × 5 rows × 4 blocks = 2500 m^2^) for a total of 5000 m^2^. From 1 March 2021 to 31 October 2021, a defined linear transect of 25 m each in eight replicates (25.8 = 200 m) was used for the assessments of the beneficial insects along the row.

Specimens were collected with the net technique, from 10:00 a.m. to 4:00 p.m., on flowers (pollinators) and vegetative organs (predators) of the spontaneous and planted (intercropping) plant species. All specimens were transferred in the laboratory, dry prepared, and identified, when necessary, through the observation of sexual structures. The month of collection, number of specimens, and visited plants are given for all species. Specimens of wild bees were identified using the taxonomic keys in [75,76,77], as Lepidoptera [78], Diptera Syrphidae [38], Coleoptera Coccinellidae [79,80], and Neuroptera [81]. The classification followed Michener [47] for supra-specific taxa, and their nomenclature was according to [82,83]. The examined specimens were preserved in the collections of the authors and in the entomological collection of CREA-OFA of Acireale.

### 4.4. Spontaneous Flora Assessment and Analysis

Weed abundance and community composition and diversity were evaluated and monitored twice during the experiment: at the start of spring on 25 March 2021 and in autumn on 6 October 2021 at day of the year (DOY) 141 and 255, respectively, corresponding to the stages of maximum development of the natural cover (i.e., spring and autumn). At each sampling stage, weed cover (i.e., the percentage of the surface area of the quadrat covered by weeds) was evaluated at a species level by randomly placing three 1.0 m^2^ quadrats within each block per soil management in the inter-row (3 squares × 4 subplots × 2 soil managements = 24) and three 1.0 m^2^ quadrats for each intercropping species in each intra-row, in all blocks for each soil management (3 squares × 5 consociated species or control × 4 subplots × 2 soil managements = 120). Density was evaluated by placing two 0.60 × 0.60 m^2^ quadrats in the intra-row space and four 0.25 × 0.25 m^2^ quadrats in each soil management system per block. Cover and density assessment allowed providing the total cover (%) and the total density of the community.

### 4.5. Tree Growth Monitoring

Biometrical measurements of the young olive trees were conducted on 15 December 2020 and on 15 October 2021, and the relative increments were calculated. Measurements regarded the total height of the tree, the widths of the canopy (in two perpendicular directions from the projection on the ground at noon), and the canopy height, measured from the first primary branch insertion point to the top. The canopy volume was calculated assuming an elliptical shape [84]. The trunk cross-sectional area (TCSA) was calculated from the trunk circumference measured at 20 cm from the ground.

Pruning was realized on 15 February 2020, and the weight of the removed material was recorded, while the weight per tree of new emitted suckers was recorded in October 2021.

Moreover, the total vegetative growth was obtained by measuring the length improvement from the beginning of the vegetative growth (15 April 2021) to the end of the experiment (31 October 2021) of two 1 year old mixed shoots per plants, randomly selected and labeled around the canopy of the trees at 1.0–1.2 m height from the ground.

### 4.6. Statistical Analysis

Analysis of variance (ANOVA) was performed with Jamovi 2.0.0 statistical software (The jamovi project, 2021). One-way analysis of variance (ANOVA) was carried out on the differences among the canopy treatments. A post hoc analysis based on Tukey’s HSD test (Tukey’s honestly significant difference) was performed at a significance level (*p*-value) of 0.05, 0.01, and 0.001, respectively. Principal component analysis (PCA) was performed with Past 4.03 statistical software (Oyvind Hammer), to assess the effect of the ASC along the row, as well as the role of tillage used in the inter-row soil management in the development, abundance, and distribution of the weed community in spring and in autumn.

## 5. Conclusions

The obtained results, even if preliminary, evidence the role of diversification strategies in recovering rather than halting the loss of wild biodiversity in agricultural fields. In particular, the agronomical techniques proposed for the young organic olive, have been shown to be an evaluable option for promoting the presence of pollinators and, thus, supporting the potential production. The inter-row management resulted in a diversified spontaneous flora community, more service provider than competitor. In addition, the wild plants on the row had a sheltering effect on the living mulch species during the hot period, demonstrating a flow of services between the components of the agroecosystem. Among the studied living mulch species, sage and lemongrass were able to create an almost continuous hedge along the row and a semi-full soil cover, thus reducing the need for weed management in the intra-row soil strip and improving the beneficial insects without influencing the plant growth.

In a nutshell, current results indicated that the agroecological practices adopted increase the richness of the biota and, hence, the complexity of the Arthropod fauna in terms of number of species and taxonomic complexity. The knowledge of the two groups of insects investigated is of primary importance for evaluating the local populations of pollinators and predators of wild and cultivated plants.

## Figures and Tables

**Table 1:** Hymenoptera Apoidea, Lepidoptera, Diptera, Neuroptera, and Coleoptera collected in the years 2020–2021 in the field inter-rows, and in the year 2021 in the consociated rows. * In this species, the larvae are predators.

Order	Family	Species	Wild Plants in the Inter-Rows	Consociated Plants in the Row
Pollinators				
Hymenoptera	Colletidae	*Hylaeus cornutus* Curtis, 1831	*Foeniculum vulgare*	*Helichrysum italicum*
	Andrenidae	*Andrena aerinifrons* Dours, 1873	*Sinapis arvensis*	
			*Ranunculus muricatus*	
		*Andrena bicolorata* (Rossi, 1790)	*Sinapis arvensis*	
		*Andrena pilipes* Fabricius, 1781	*Senecio vulgaris*	
		*Andrena brumanensis* Friese, 1899	*Ranunculus muricatus*	*Salvia officinalis*
		*Andrena distinguenda* Schenck, 1871	*Glebionis coronaria*	
		*Andrena labialis* (Kirby, 1802)	*Ecballium elaterium*	
		*Andrena nigroaenea* (Kirby, 1802)	*Sinapis arvensis*	
	Halictidae	*Halictus fulvipes* (Klug, 1817)	*Galactites tomentosa*	*Thymus vulgaris*
		*Halictus quadricinctus* (Fabricius, 1776)	*Senecio vulgaris*	
		*Halictus scabiosae* (Rossi, 1790)	*Senecio vulgaris* *Dittrichia viscosa*	*Thymus vulgaris*
		*Lasioglossum malachurum* (Kirby, 1802)	*Ecballium elaterium*	*Helichrysum italicum*
	Megachilidae	*Heriades rubicola* Pérez, 1890	*Dittrichia viscosa*	*Helichrysum italicum*
		*Osmia latreillei* (Spinola, 1806)	*Glebionis coronaria*	*Salvia officinalis*
		*Osmia signata* Erichson, 1835	*Glebionis coronaria*	
		*Rhodanthidium siculum* (Spinola, 1838)	*Oxalis pes-caprae*	*Salvia officinalis*
		*Megachile sicula* (Rossi, 1792)	*Galactites tomentosa*	
	Apidae	*Xylocopa violacea* (Linnaeus, 1758)	-	*Salvia officinalis* *Thymus vulgaris*
		*Ceratina cyanea* Kirby, 1802	*Ecballium elaterium*	*Helichrysum italicum*
		*Nomada discrepans* Schmiedeknecht, 1882	*Sinapis arvensis*	
		*Nomada distinguenda* Morawitz, 1874	*Raphanus raphanistrum*	
		*Eucera algira* Brullé, 1840	*Raphanus raphanistrum*	
		*Eucera eucnemidea* Dours, 1873	*Galactites tomentosa*	
		*Eucera nigrescens* Pérez, 1879	*Glebionis coronaria*; *Vicia* sp.	
		*Eucera nigrilabris* Lepeletier, 1841	*Raphanus raphanistrum*	
		*Eucera numida* Lepeletier, 1841	*Vicia sativa*	
		*Eucera oraniensis* Lepeletier, 1841	*Glebionis coronaria*	*Salvia officinalis*
			*Galactites tomentosa*	
		*Amegilla garrula* (Rossi, 1790)	-	*Salvia officinalis* *Thymus vulgaris*
		*Amegilla quadrifasciata* (de Villers, 1789)	-	*Salvia officinalis* *Thymus vulgaris*
		*Anthophora dispar* Lepeletier, 1841	*Fumaria officinalis*	*Salvia officinalis*
		*Anthophora plumipes squalens* Dours, 1869	*Fumaria officinalis* *Papaver rhoeas*	
		*Bombus pascuorum siciliensis* Tkalcu, 1977	*Vicia sativa*	*Salvia officinalis* *Thymus vulgaris*
		*Bombus terrestris* (Linnaeus, 1758)	*Vicia sativa*	*Salvia officinalis* *Thymus vulgaris*
Lepidoptera	Sphingidae	*Macroglossum stellatarum* (Linnaeus, 1758)	*Convolvulus arvensis*	*Thymus vulgaris*
		*Hyles euphorbiae* (Linnaeus, 1758)	-	
		*Hyles livornica* (Esper, 1780)	*Convolvulus arvensis*	*Helichrysum italicum*
	Sesiidae	*Tinthia tineiformis* (Esper, 1789)	*Convolvulus arvensis*	
	Geometridae	*Rhodometra sacraria* (Linnaeus, 1767)	-	*Thymus vulgaris*
		*Menophra abruptaria* (Thunberg, 1792)	*Dittrichia viscosa*	*Thymus vulgaris*
	Noctuidae	*Heliothis peltigera* (Denis & Schiffermüller, 1775)	*Senecio vulgaris*	*Helichrysum italicum* *Thymus vulgaris*
		*Autographa gamma* (Linnaeus, 1758)		*Salvia officinalis*
	Hesperiidae	*Carcharodus alceae* (Esper, 1780)	*Lysimachia arvensis*	*Thymus vulgaris*
	Lycaenidae	*Lycaena alciphron* (Rottemburg, 1775)	*Althaea officinalis*	*Thymus vulgaris*
		*Lycaena phlaeas* (Linnaeus, 1761)	*Polygonum aviculare*	*Thymus vulgaris*
			*Portulaca oleracea*	
			*Ranunculus muricatus*	
	Nymphalidae	*Aglais urticae* (Linnaeus, 1758)	*Althaea officinalis*	*Helichrysum italicum*
		*Vanessa atalanta* (Linnaeus, 1758)	*Althaea officinalis* *Convolvulus arvensis* *Ecballium elaterium*	*Salvia officinalis* *Thymus vulgaris*
		*Vanessa cardui* (Linnaeus, 1758)	*Ranunculus muricatus*	*Thymus vulgaris*
		*Lasiommata megera* (Linnaeus, 1767)	*Polygonum aviculare*	*Salvia officinalis* *Thymus vulgaris*
		*Pararge aegeria* (Linnaeus, 1758)	-	*Thymus vulgaris*
	Papilionidae	*Iphiclides podalirius* (Linnaeus, 1758)	-	*Helichrysum italicum*
		*Papilio machaon* Linnaeus, 1758	*Dittrichia viscosa*	
	Pieridae	*Colias croceus* (Geoffroy, 1785)	*Ecballium elaterium*	*Thymus vulgaris*
		*Gonepteryx cleopatra* (Linnaeus, 1767)	-	*Thymus vulgaris*
		*Pieris brassicae* (Linnaeus, 1758)	*Capsella bursa-pastoris*	*Thymus vulgaris*
			*Raphanus raphanistrum*	
			*Sinapis arvensis*	
		*Pieris mannii* (Mayer, 1851)	*Beta vulgaris*	
		*Pieris rapae* (Linnaeus, 1758)	*Portulaca oleracea* *Sinapis arvensis*	*Thymus vulgaris*
Diptera	Syrphidae	*Episyrphus balteatus* (DeGeer, 1776) *	-	*Thymus vulgaris*
		*Eupeodes luniger* (Meigen, 1822) *	*Ranunculus muricatus*	
		*Eristalinus taeniops* (Wiedemann, 1818)	*Beta vulgaris* *Polygonum aviculare*	*Thymus vulgaris*
		*Eristalis tenax* (Linnaeus, 1758)	-	*Helichrysum italicum*
				*Thymus vulgaris*
		*Syritta pipiens* (Linnaeus, 1758)	*Portulaca oleracea*	
Predators				
Neuroptera	Chrysopidae	*Chrysopa viridana* Schneider, 1845	-	*Thymus vulgaris*
		*Chrysoperla carnea* (Stephens, 1836)	*Ranunculus muricatus*	*Salvia officinalis*
			*Beta vulgaris*	*Helichrysum italicum*
Coleoptera	Coccinellidae	*Chilocorus bipustulatus* (Linnaeus, 1758)	*Ecballium elaterium*	*Thymus vulgaris*
		*Coccinella septempunctata* Linnaeus, 1758	*Amaranthus retroflexus*	*Helichrysum italicum*
			*Diplotaxis erucoides*	*Salvia officinalis*
		*Hippodamia variegata* (Goeze, 1777)	*Cerinthe major*	-
		*Propylea quatuordecimpunctata* (Linnaeus, 1758)	*Althaea officinalis*	-
		*Scymnus interruptus* (Goeze, 1777)	*Salvia officinalis*	*Senecio vulgaris*
		*Scymnus subvillosus* (Goeze, 1777)	*Diplotaxis erucoides*	

**Table 2:** Seasonal presence of Apoidea species in the experimental farm of Palazzelli during the years 2020–2021.

Hymenoptera		Jan	Feb	Mar	Apr	May	Jun	Jul	Aug	Sep	Oct	Nov	Dec	Years	
														2020	2021
	Colletidae														
1	*Hylaeus cornutus*							√						X	
	Andrenidae														
2	*Andrena aerinifrons*			√											X
3	*Andrena bicolorata*				√									X	
4	*Andrena brumanensis*					√									X
5	*Andrena distinguenda*		√	√										X	X
6	*Andrena labialis*					√		√						X	X
7	*Andrena nigroaenea*		√	√										X	X
8	*Andrena pilipes*				√	√								X	X
	Halictidae														
9	*Halictus fulvipes*					√	√	√		√				X	X
10	*Halictus quadricinctus*						√	√	√					X	X
11	*Halictus scabiosae*					√	√	√	√					X	X
12	*Lasioglossum malachurum*			√	√										X
	Megachilidae														
13	*Heriades rubicola*							√	√	√	√			X	X
14	*Osmia latreillei*				√	√								X	X
15	*Osmia signata*						√							X	
16	*Rhodanthidium siculum*			√	√									X	X
17	*Megachile sicula*		√	√										X	X
	Apidae														
18	*Xylocopa violacea*		√	√	√	√	√			√	√			X	X
19	*Ceratina cyanea*						√	√						X	X
20	*Nomada discrepans*								√					X	
21	*Nomada distinguenda*								√						X
22	*Eucera algira*		√	√											X
23	*Eucera eucnemidea*				√	√	√							X	X
24	*Eucera nigrescens*		√	√	√									X	X
25	*Eucera nigrilabris*				√										X
26	*Eucera numida*				√	√								X	X
27	*Eucera oraniensis*		√	√	√									X	X
28	*Amegilla garrula*					√	√							X	X
29	*Amegilla quadrifasciata*						√	√	√					X	X
30	*Anthophora dispar*	√	√	√	√									X	X
31	*Anthophora plumipes squalens*		√	√	√	√								X	X
32	*Bombus pascuorum siciliensis*						√	√	√	√	√			X	X
33	*Bombus terrestris*	√	√	√	√	√	√			√	√			X	X

**Table 3:** List of the spontaneous flora species detected in spring and in autumn in both the inter-row and the intra-row of the experimental field ‘long-term trial on organic olive (BiOlea)’ at Palazzelli.

Spontaneous Flora Species	Family	EPPO Code	Spring			Autumn			
			Inter-Row		Intra-Row	Inter-Row		Intra-Row	
			Zero Tillage	Minimum Tillage		Zero Tillage	Minimum Tillage		
*Amaranthus retroflexus* L.	Amaranthaceae	AMARE	+	+	+	+	+	+	
*Arum maculatum* L.	Araceae	ABGMA	-	-	+	-	-	-	
*Avena sterilis* L.	Poaceae	AVEST	+	-	+	-	-	-	
*Beta vulgaris* L.	Chenopodiaceae	BEAVX	+	+	+	+	+	+	
*Brassica nigra* (L.) W.D.J. Koch	Brassicaceae	BRSNI	-	-	+	+	+	-	
*Capsella bursa-pastoris* (L.) Medik.	Brassicaceae	CAPBP	-	-	+	+	+	-	
*Convolvolus arvensis* L.	Convolvulaceae	CONAR	-	+	+	+	+	-	
*Cynodon dactylon* (L.) Pers.	Poaceae	CYNDA	+	+	+	+	+	+	
*Cyperus rotundus* L.	Cyperaceae	CYPRO	+	+	+	+	+	+	
*Dactylis glomerata* L.	Poaceae	DACGL	+	-	+	-	-	-	
*Digitaria sanguinalis* (L.) Scop.	Poaceae	DIGSA	-	-	+	-	-	+	
*Dittrichia viscosa* (L.) Greuter	Asteraceae	INUVI	-	-	+	-	-	-	
*Ecballium elaterium* (L.) A. Rich.	Cucurbitaceae	ECBEL	-	-	+	-	-	+	
*Elymus repens* (L.) Gould	Poaceae	AGGRE	-	-	-	+	+	-	
*Erigeron canadensis* L.	Asteraceae	ERICA	-	-	-	-	-	+	
*Euphorbia prostrata* Aiton	Euphorbiaceae	EPHPT	-	-	-	-	-	+	
*Fumaria officinalis* L.	Papaveraceae	FUMOF	+	+	+	+	-	-	
*Lactuca sativa* subsp. *serriola* (L.) Galasso, Banfi, Bartolucci & Ardenghi	Asteraceae	LACSE	+	+	+	-	-	-	
*Lamium amplexicaule* L.	Lamiaceae	LAMAM	-	-	-	-	-	+	
*Lolium perenne* L.	Poaceae	LOLPE	+	+	+	-	-	-	
*Lysimachia arvensis* (L.) U. Manns & Anderb.	Primulaceae	LYSAR	-	-	+	-	-	-	
*Malva sylvestris* L.	Malvaceae	MALSY	-	-	+	+	+	-	
*Myosotis arvensis* (L.) Hill	Boraginaceae	MYOAR	-	-	+	-	-	-	
*Oxalis pes-caprae* L.	Oxalidaceae	OXAPC	-	-	-	-	-	+	
*Papaver rhoeas* L.	Papaveraceae	PAPRH	+	+	+	-	-	-	
*Polygonum aviculare* L.	Polygonaceae	POLAV	+	+	+	+	+	+	
*Portulaca oleracea* L.	Portulacaceae	POROL	+	+	+	+	+	+	
*Ranunculus muricatus* L.	Ranunculaceae	RANMU	-	-	+	-	-	-	
*Raphanus raphanistrum* L.	Brassicaceae	RAPRA	-	-	+	+	+	+	
*Senecio vulgaris* L.	Asteraceae	SENVU	-	-	+	-	-	+	
*Setaria verticillata* (L.) P. Beauv.	Poaceae	SETVE	-	-	-	+	+	+	
*Sinapis arvensis* L.	Brassicaceae	SINAR	-	-	+	-	-	-	
*Solanum nigrum* L.	Solanaceae	SOLNI	-	-	-	-	-	+	
*Sonchus asper* subsp. *glaucescens* (Jord.) Ball	Asteraceae	SONAR	-	-	-	-	-	+	
*Sonchus oleraceus* L.	Asteraceae	SONOL	-	-	-	-	-	+	
*Stellaria media* (L.) Vill.	Caryophyllaceae	STEME	-	-	-	-	-	+	
*Triticum* spp.	Poaceae	-	-	-	+	-	-	-	
*Urtica dioica* L.	Urticaceae	URTDI	-	+	+	-	-	-	
*Veronica peregrina* L.	Plantaginaceae	VERPG	-	-	-	-	-	+	
Total richness (No. species)			12	12	28	14	13	20	

**Table 4:** (**A**,**B**) Principal component analysis (PCA) eigenvalues and percentage variance of the studied samples from experimental trial in relation to the inter-row (**A**) and intra-row (**B**) management in spring.

A			B		
PC	Eigenvalue	% Variance	PC	Eigenvalue	% Variance
1	3.36	20.97	1	3.02	11.17
2	2.51	15.71	2	2.38	8.80
3	2.41	15.08	3	2.15	7.95
4	1.78	11.13	4	1.87	6.93
5	1.30	8.15	5	1.53	5.67
6	1.25	7.80	6	1.49	5.50
7	1.19	7.41	7	1.39	5.17
8	0.68	4.24	8	1.26	4.67
9	0.55	3.42	9	1.13	4.17
10	0.44	2.77	10	1.12	4.14
11	0.26	1.60	11	1.04	3.86
12	0.20	1.23	12	1.01	3.76
13	0.05	0.29	13	0.94	3.49
14	0.02	0.13	14	0.89	3.30
15	0.01	0.07	15	0.85	3.16
			16	0.77	2.86
			17	0.71	2.62
			18	0.65	2.39
			19	0.58	2.14
			20	0.52	1.93
			21	0.38	1.42
			22	0.35	1.30
			23	0.26	0.97
			24	0.23	0.84
			25	0.19	0.69
			26	0.16	0.58
			27	0.13	0.50

**Table 5:** Influence of soil management strategy on olive tree growth in pre-growing season on 15 December 2020 as compared with plant growth in autumn on 15 October 2021 and percentage increase. Means indicated by different letters are significantly different (lowercase *p* ≤ 0.05, ±standard deviation) according to Tukey’s HSD test, for each treatment and parameter. ^ns^ = not significant.

	15 December 2020			15 October 2021			Percentage Increase (Δ%)		
Treatment	Trunk Cross-Sectional Area (cm^2^)	Canopy Height (cm)	Canopy Volume (m^3^)	Trunk Cross-Sectional Area (cm^2^)	Canopy Height (cm)	Canopy Volume (m^3^)	Trunk Cross-Sectional Area (Δ%)	Canopy Height (Δ%)	Canopy Volume (Δ%)
Nocellara etnea—minimum tillage	6.32 ± 2.4 ^ab^	103.9 ± 22.06 ^a^	0.29 ± 0.11 ^ns^	13.7 ± 2.69 ^b^	152.6 ± 23.43 ^ns^	1.55 ± 0.38 ^a^	117	146	542
Nocellara del Belice—minimum tillage	4.99 ± 2.09 ^b^	72.5 ± 18.65 ^b^	0.21 ± 0.09 ^ns^	12.4 ± 2.74 ^b^	105.8 ± 22.31 ^ns^	0.73 ± 0.19 ^b^	148	145	339
Nocellara etnea—zero tillage	8.87 ± 2.03 ^a^	82.6 ± 31.68 ^ab^	0.32 ± 0.12 ^ns^	18.2 ± 2.23 ^a^	143.1 ± 29.29 ^ns^	1.23 ± 0.22 ^a^	205	173	387
Nocellara del Belice—zero tillage	4.13 ± 2.09 ^ab^	82.13 ± 21.65 ^ab^	0.34 ± 0.10 ^ns^	8.1 ± 3.87 ^b^	120.5 ± 28.20 ^ns^	1.05 ± 0.19 ^b^	196	146	438

**Table 6:** Main soil physical and chemical properties at the experimental field ‘long-term trial on organic olive (BiOlea)’.

Parameter	Unit Measure	Value
Sand	%	60
Silt	%	21
Clay	%	19
pH		7.8
Electrical conductivity (1:2.5)	dS/m	0.26
Organic matter	%	2.69
Total nitrogen (N)	‰	0.140
Exchangeable phosphorus (P)	ppm P	53
Exchangeable potassium (K)	ppm K	3628
Cation exchange capacity (CEC)	meq/100 g	64.98

## Data Availability

All data are available via email request to the corresponding authors.
